# The association of fear of falling and physical and mental Health-Related Quality of Life (HRQoL) among community-dwelling older persons; a cross-sectional study of Urban Health Centres Europe (UHCE)

**DOI:** 10.1186/s12877-023-04004-y

**Published:** 2023-05-13

**Authors:** Sophie (S. A.) Korenhof, Amy (A.) van Grieken, Carmen (C. B.) Franse, Siok Swan (S. S.) Tan, Arpana (A.) Verma, Tamara (T.) Alhambra, Hein (H.) Raat

**Affiliations:** 1 Department of Public Health, Erasmus University Medical Center, PO Box 2040, 3000 CA Rotterdam, the Netherlands; 2 Inholland University of Applied Sciences, Rotterdam, the Netherlands; 3 Manchester Urban Collaboration On Health, Centre for Epidemiology, Division of Population Health, Health Services Research and Primary Care, Manchester Academic Health Science Centre, The University of Manchester, Manchester, UK; 4 Polibienestar Research Institute, University of Valencia, Valencia, Spain

**Keywords:** Fear of falling, Health-related quality of life, Community-dwelling older adults, Accidental falls, Quality of life, Aged 80 and over, Independent living, Europe

## Abstract

**Background:**

The share of people over 80 years in the European Union is estimated to increase two-and-a-half-fold from 2000 to 2100. A substantial share of older persons experiences fear of falling. This fear is partly associated with a fall in the recent past. Because of the associations between fear of falling, avoiding physical activity, and the potential impact of those on health, an association between fear of falling and low health-related quality of life, is suggested. This study examined the association of fear of falling with physical and mental Health-Related Quality of Life (HRQoL) among community-dwelling older persons in five European countries.

**Methods:**

A cross-sectional study was conducted using baseline data of community-dwelling persons of 70 years and older participating in the Urban Health Centers Europe project in five European countries: United Kingdom, Greece, Croatia, the Netherlands and Spain. This study assessed fear of falling with the Short Falls Efficacy Scale-International and HRQoL with the 12-Item Short-Form Health Survey. The association between low, moderate or high fear of falling and HRQoL was examined using adjusted multivariable linear regression models.

**Results:**

Data of 2189 persons were analyzed (mean age 79.6 years; 60.6% females). Among the participants, 1096 (50.1%) experienced low fear of falling; 648 (29.6%) moderate fear of falling and 445 (20.3%) high fear of falling. Compared to those who reported low fear of falling in multivariate analysis, participants who reported moderate or high fear of falling experienced lower physical HRQoL (β = -6.10, *P* < 0.001 and β = -13.15, *P* < 0.001, respectively). In addition, participants who reported moderate or high fear of falling also experienced lower mental HRQoL than those who reported low fear of falling (β = -2.31, *P* < 0.001 and β = -8.80, *P* < 0.001, respectively).

**Conclusions:**

This study observed a negative association between fear of falling and physical and mental HRQoL in a population of older European persons. These findings emphasize the relevance for health professionals to assess and address fear of falling. In addition, attention should be given to programs that promote physical activity, reduce fear of falling, and maintain or increase physical strength among older adults; this may contribute to physical and mental HRQoL.

**Supplementary Information:**

The online version contains supplementary material available at 10.1186/s12877-023-04004-y.

## Introduction

Europe holds a relatively high proportion of persons over 65 years old [1]. It is estimated that the share of those aged 80 years or older in the European Union will increase two–and-a-half-fold between 2020 and 2100, i.e. from 5.9% to 14.6% [2]. Approximately 30% of community-dwelling persons over 65 years of age experienced a fall during the past year [3–6]. Related to this, a substantial part of community-dwelling older persons experience fear of falling [1, 5–8]. As reported in the literature, the prevalence of fear of falling ranges from 3 to 85% [6, 7, 9]. The reported prevalence depends on the specific study sample, the number of previous falls in the study population, and the specific definition or measurement tool used [6, 7, 9–11].

Fear of falling is defined as the ongoing concern about falling that could ultimately lead to activity avoidance [12]. And is also associated with incident disability [13] and increased mortality as well [14]. Fear of falling is suggested to have a bidirectional relationship with falling itself; fear of falling may be a predictor of falling, and vice versa; falling has been reported to be associated with subsequent fear of falling [15]. In a longitudinal study by Friedman et al., of the participants without fear of falling at baseline, persons who experienced a fall were twice as likely to experience fear of falling at follow-up compared to persons who had not experienced a fall [15]. Persons who experience fear of falling may develop an aversion regarding physical activity behaviors, leading to a decline in physical health [15]. Therefore, fear of falling is considered a risk factor for future falls [6, 15, 16]. In addition, fear of falling also showed a positive association with frailty [17].

Health-related quality of life (HRQoL) is considered an important outcome on healthy ageing in geriatric medicine and public health [18]. HRQoL is defined as a multidimensional and subjective measure of well-being in relation to health, embodying the appraisal of physical, mental and social well-being [19, 20]. Because of the associations between fear of falling, tendency to avoid physical activity, and potential impact of those on health, an association between fear of falling and low health-related quality of life, is suggested [9]. Landi et al. have shown that disability is an important influence for risk of death in older people [21]. In addition, Schoene et al. have recommended studying the impact of fear of falling on HRQoL in ageing populations [22]. Nevertheless, thus far, only a few studies have studied these associations [8, 22]. Insight into the association between fear of falling and HRQoL may inform healthcare professionals on the need to support older persons in maintaining healthy [22].

This study aimed to investigate the associations between fear of falling and HRQoL, corrected for previous falls, among community-dwelling older persons aged 70 years and over in Europe. We explored interaction by age, gender, country, level of education, living situation, physical activity behaviors, experienced falls in the previous 12 months and multi-morbidity. Following the literature, the hypothesis was that the association between fear of falling and HRQoL would be observed among participants that were women [9, 23], older [9], lower educated [9, 24], living alone [24] and physically active once or less per week [23, 25]. With regard to country, we hypothesized that there may be a different association between countries [26].

## Methods

### Study design

This cross-sectional study used baseline data from the Urban Health Centres Europe (UHCE) project for secondary analysis. The evaluation design and effectiveness of the UHCE study, a study regarding anticipatory preventive care for older persons, were described by Franse et al. (2017) [26, 27]. This project was implemented in five European cities (Greater Manchester, United Kingdom; Pallini, Greece; Rijeka, Croatia; Rotterdam, the Netherlands; and Valencia, Spain). 2325 UHCE participants were enrolled between May 2015 and June 2017. Participants’ inclusion criteria were: aged 70 years and older, community-dwelling and able to participate in the study for at least six months, according to their physician. Exclusion criteria were: not fluent in the local language and unable to cognitively assess the risks and benefits of participation. Data were obtained by self-reported questionnaires. All participants provided written informed consent. Ethical committee procedures have been followed in all cities and institutions involved, and approval has been provided [27].

The current study used baseline data (2325 participants) from the UHCE project. Participants with missing data on age, gender, fear of falling or HRQoL were excluded from the analyses (*N* = 136). Thus, the population for analysis consisted of 2189 participants (see Suppl. Figure 1).

### Measures

#### Fear of falling

Fear of falling was assessed by the Falls Efficacy Scale International Short (Short FES-I), which has been shown to have adequate validity in European populations (Cronbach’s alpha 0.92, intra-class coefficient 0.83) [28–30]. The Short FES-I assesses fear of falling concerning seven activities: going up and down stairs, getting dressed or undressed, taking a bath or shower, getting in or out of a chair, going up or down stairs, reaching for something above your head or on the ground, walking up or down a slope and going out to a social event; and has four possible answer categories: not at all concerned (1 point), somewhat concerned (2 points), fairly concerned (3 points) and very concerned (4 points). The final scores of the Short FES-I range between 7 and 28, whereby a score of 7–8 corresponds to low fear of falling, and scores of 9–13 and 14–28 correspond to moderate and high fear of falling, respectively [29].

#### Health-Related Quality of Life (HRQoL)

The 12-Item Short Form Health Survey (SF-12) version 2 was used to assess physical and mental HRQoL; it has shown to be a valid instrument for assessing HRQoL in various European countries in adult populations [31, 32]. The questionnaire consists of eight domains: bodily pain (1 item), vitality (1), physical functioning (2), physical role functioning (2), emotional role functioning (2), mental health (2), social functioning (1) and general health (1). The raw SF-12 scores were first transformed to provide scores ranging from 0 (the worst) to 100 (the best). After that, the physical and mental component summary scores (PCS and MCS) were calculated; these are standard scores with a mean value of 50 points and a standard deviation of 10 points [31].

### Covariates

#### Socio-demographic factors

The socio-demographic factors age, gender, country, level of education and living situation (alone/ not alone) were assessed. The level of education was divided into three groups according to the International Standard Classification of Education (ISCED). ISCED level 0–1 was defined as ‘primary or lower’; ISCED level 2–5 was defined as ‘secondary’, and ISCED level 6–8 was defined as ‘tertiary’ [33].

#### Lifestyle and general health factors

Alcohol risk (yes/ no), physical activity (once a week or less/ more than once a week), smoking (yes/ no) and multi-morbidity (< 2 or ≥ 2 chronic conditions) were assessed [34]. Smoking was assessed with one item that asked whether a person smoked at present. Alcohol risk was assessed with the AUDIT-C, which contains three items to screen high-risk alcohol use on a scale from 0 (lowest risk) to 12 (highest risk) [35]. A score of at least 4 for men and 3 for women is considered hazardous drinking or active alcohol use disorder and therefore defined as ‘alcohol risk’ [35]. Multi-morbidity was defined as having (had) at least two out of 14 chronic conditions [36]: heart attack, hypertension, diabetes, stroke, high blood cholesterol, asthma, arthritis, osteoporosis, chronic lung disease, cancer or malignant tumor, stomach or duodenal ulcer, Parkinson’s disease, cataract and hip fracture or femoral fracture [37].

### Statistical analyses

Descriptive statistics of the covariates, socio-demographic and lifestyle factors were determined and stratified by fear of falling category. A one-way analysis of variance (ANOVA) test for continuous variables (age) and Pearson’s chi-squared tests for categorical variables (all other variables) was conducted to compare the low, moderate and high fear of falling groups with each other on the covariates. Differences in HRQoL scores were compared between participants with moderate or high and low fear of falling. Cohen’s effect size estimations (*d*) were calculated, which relate the difference in mean scores to the dispersion of the scores: [Mean_1_ − Mean_2_]/(σ_pooled_); where pooled standard deviation was calculated as σ_pooled_ = √[(σ_1_^2^ + σ_2_^2^) / 2]. Following Cohen's suggested guidelines, 0.20 ≤ *d* < 0.50 was taken to indicate a small effect size, 0.50 ≤ *d* < 0.80 a moderate effect size, and *d* ≥ 0.8 a large effect size [38].

Age, gender, country, level of education, living situation, alcohol risk, physical activity, smoking, and multi-morbidity of included versus excluded participants were compared via Pearson’s chi-squared tests for categorical variables and *t*-tests for continuous variables. A *P-*value of < 0.05 was considered significant.

Multivariable linear regression analyses were performed to examine the associations between fear of falling and physical and mental HRQoL. First, associations were tested for fear of falling (low, moderate, high) and physical or mental HRQoL (crude model). Second, socio-demographic and lifestyle factors were added to this model (adjusted model). Beta coefficients were calculated for; and presented with 95%-confidence intervals (CIs). A *P*-value of < 0.05 was considered significant. Similarly, the association was also tested between continuous fear of falling scores and physical and mental HRQoL. (Suppl. Table 1).

We performed linearity tests, normality of residuals with kernel density plots and tests for multicollinearity with variance inflation factors.

Finally, the interaction between the fear of falling score (continuous) and age, gender, country, level of education, living situation, alcohol risk, physical activity and multi-morbidity was evaluated by adding interaction terms to the adjusted models (Suppl. Table 2). Models were run separately for mental and physical HRQoL. The 2-sided significance threshold, after Bonferroni correction for multiple testing, was set at 0.05 (original *P*-value) / 18 (number of tests performed) = *P* = 0.0028. Stratified linear regression analyses were run for statistically significant interaction terms. (Suppl. Tables 3, 4 and 5).

All analyses were performed with SPSS version 25.0 (IBM SPSS Statistics for Windows, IBM Corp.: Armonk, NY, USA).

## Results

The mean age of the participants was 79.7 (SD 5.6) years; 60.6% were female. Overall, 50.1% reported a ‘low’, 29.6% a ‘moderate’, and 20.3% a ‘high’ level of fear of falling; the mean score on the Short FES-I was 10.6 (SD 4.9). The mean physical and mental HRQoL scores were 41.8 (SD 12.1) and 50.2 (SD 10.7), respectively. Mean physical and mental HRQoL scores were lower in the subgroups who reported ‘moderate’ and ‘high’ fear of falling compared to those who reported ‘low’ fear of falling (*P* < 0.001). The percentage of participants who experienced a fall in the last 12 months was higher in the subgroup who reported ‘high’ or ‘moderate’ fear of falling compared to those who reported ‘low’ fear of falling. Cohen’s *d* effect size between participants of the ‘low’ and ‘high’ fear of falling subgroups for physical and mental HRQoL was calculated at *d* ≥ 0.80. (Table 1).

**Table 1:** General characteristics of the study population (*n* = 2189)

		**Fear of falling**			*P*-value
	Total (*N* = 2189)	Low (*N* = 1096)	Moderate (*N* = 648)	High (*N* = 445)	
**Age (SD)**	79.7 (5.6)	78.8 (5.5)	80.0 (5.6)	81.3 (5.6)	< 0.001
**Gender**					
Female (%)	1326 (60.6)	550 (50.2)	443 (68.4)	333 (74.8)	< 0.001
Male (%)	863 (39.4)	546 (49.8)	205 (31.6)	112 (25.2)	
**Country**					< 0.001
United Kingdom (%)	536 (24.5)	333 (30.4)	134 (20.7)	69 (15.5)	
Greece (%)	334 (15.3)	138 (12.6)	115 (17.7)	81 (18.2)	
Croatia (%)	485 (22.2)	109 (9.9)	166 (25.6)	210 (47.2)	
The Netherlands (%)	339 (15.5)	217 (19.8)	85 (13.1)	37 (8.3)	
Spain (%)	495 (22.6)	299 (27.3)	148 (22.8)	48 (10.8)	
**Level of education **^**a**^					< 0.001
Primary or less (%)	593 (27.4)	269 (24.9)	206 (32.1)	118 (26.8)	
Secondary or equivalent (%)	1357 (62.7)	675 (62.4)	381 (59.4)	301 (68.4)	
Tertiary or higher (%)	213 (9.8)	138 (12.8)	54 (8.4)	21 (4.8)	
**Living situation **^**a**^					< 0.001
Living with others (%)	1358 (62.2)	726 (66.2)	397 (61.6)	235 (53.0)	
Living alone (%)	825 (37.8)	370 (33.8)	247 (38.4)	208 (47.0)	
**Alcohol risk **^**a**^					< 0.001
No (%)	1538 (73.8)	715 (68.0)	460 (75.4)	363 (85.8)	
Yes (%)	546 (26.2)	336 (32.0)	150 (24.6)	60 (14.2)	
**Physical activity **^**a**^					< 0.001
= < once a week (%)	617 (28.4)	162 (14.8)	185 (28.8)	270 (61.4)	
> once a week (%)	1558 (71.6)	931 (85.2)	457 (71.2)	170 (38.6)	
**Smoking **^**a**^					0.184
Yes (%)	158 (7.2)	78 (7.1)	55 (8.5)	25 (5.6)	
No (%)	2026 (92.8)	1017 (97.8)	589 (91.5)	420 (94.4)	
**Multi-morbidity **^**a**^					< 0.001
Yes (%)	1988 (90.9)	954 (87.0)	605 (93.5)	429 (96.4)	
No (%)	200 (9.1)	142 (13.0)	42 (6.5)	16 (3.6)	
**Fall in last 12 months **^**a**^					< 0.001
Yes (%)	667 (30.7%)	241 (22.0)	202 (31.4)	224 (51.3)	
No (%)	1506 (69.3%)	852 (78.0)	441 (68.6)	213 (48.7)	
**HRQoL **^**a**^					
PCS (SF-12; range 0–100) (SD)	41.8 (12.1)	47.6 (9.7)	40.0 (10.6)^b^	30.1 (9.6)^b^	< 0.001
MCS (SF-12; range 0–100) (SD)	50.2 (10.7)	53.4 (9.0)	50.2 (10.2)^b^	42.4 (11.5)^b^	< 0.001

Data are mean (SD) or number of participants (%)

^a^Missing items: Level of education *n* = 26, Living situation *n* = 6, alcohol risk *n* = 105, Physical activity *n* = 14, Smoking *n* = 5, Multi-morbidity *n* = 1, Fall in last 12 months *n* = 16;

Fear of falling measure, Short FES-I. *Abbreviations*: *SD* Standard deviation, *PCS* Physical Component Summary of the SF-12, *MCS* Mental Component Summary of the SF-12; SF-12, Short Form health survey, *EQ-5D-5L* EuroQol-5 Dimensions-5 level

*P*-values are based on an ANOVA test for continuous variables (age and HRQoL) and Pearson’s chi-squared tests for categorical variables (all other variables)

^b^Cohen’s d effect size compared to low-fear of falling (FoF) group: PCS Moderate FoF, *d* = 0.75; PCS High FoF, *d* = 1.81; MCS Moderate FoF, *d* = 0.33; MCS High FoF, *d* = 1.07

All variance inflation factors were lower than four, suggesting low multicollinearity. No violation of basic assumptions for regression was found.

### Comparison of included and excluded participants

Participants who were excluded (*n* = 136) were, compared to participants who were included (*n* = 2189), relatively older (80.5 versus 79.7 years old), more often smokers and more often from Greece or the Netherlands and less often from the United Kingdom, Croatia or Spain; all *P* < 0.02. Included and excluded participants showed no significant differences for the variables gender, level of education, living situation, alcohol risk, multi-morbidity, physical activity, physical HRQoL, mental HRQoL and fear of falling (see Suppl. Table 6).

### Physical HRQoL

Both the crude and adjusted linear regression models showed that participants who reported ‘moderate’ or ‘high’ fear of falling showed significantly lower physical HRQoL scores compared to participants who reported ‘low’ fear of falling (β = -6.08, 95%CI -7.05 to -5.11 and β = -13.08, 95%CI -14.29 to -11.86) for the adjusted model of ‘moderate’ or ‘high’ compared to ‘low’ fear of falling respectively; Table 2). Using the continuous fear of falling score, a higher fear of falling score was associated with lower physical HRQoL scores (Beta -0.426, *P* < 0.001) see Suppl. Table 1.

**Table 2:** Association between Fear of Falling (categorical) and physical Health-Related Quality of Life

	**Crude**	*P*-value	**Adjusted**	*P*-value
	N = 2189		N = 2040	
	Beta (95% CI)		Beta (95% CI)	
**Fear of falling (FoF)**				
FoF-low (ref.)	Ref		Ref	
FoF-moderate	-7.57 (-8.54;-6.60)	< 0.001	-6.08 (-7.05;-5.11)	< 0.001
FoF-high	-17.50 (-18.60;-16.40)	< 0.001	-13.08 (-14.29;-11.86)	< 0.001

*P*-values are based on multivariable linear regression models

Crude model: categorical fear of falling, adjusted model: crude model adjusted for age, gender, country, level of education, living situation, alcohol risk, physical activity, smoking, fall in last 12 months and multi-morbidity

*Abbreviations*: *CI* Confidence Interval, *ref*. Reference category

### Mental HRQoL

Both the crude and adjusted linear regression models showed that participants who reported ‘moderate’ or ‘high’ fear of falling reported significantly lower mental HRQoL scores compared to participants who reported ‘low fear of falling (β = -2.26, 95%CI -3.28 to -1.25 and β = -8.56, 95%CI -9.84 to -7.29) for the adjusted model of ‘moderate’ or ‘high’ compared to ‘low’ fear of falling respectively; (Table 3)**.** Using the continuous fear of falling score, a higher fear of falling score was associated with lower mental HRQoL scores (Beta -0.345,* P* < 0.001) see Suppl. Table 1.

**Table 3:** Association between Fear of Falling (categorical) and mental Health-Related Quality of Life

	**Crude**	*P*-value	**Adjusted**	*P*-value
	N = 2189		N = 2040	
	Beta (95% CI)		Beta (95% CI)	
**Fear of falling (FoF)**				
FoF-low	Ref		Ref	
FoF-moderate	-3.17 (-4.14;-2.21)	< 0.001	-2.26 (-3.28;-1.25)	< 0.001
FoF-high	-10.95 (-12.04;-9.86)	< 0.001	-8.56 (-9.84;-7.29)	< 0.001

*P*-values are based on multivariable linear regression models

Crude model: categorical fear of falling, adjusted model: crude model adjusted for age, gender, country, level of education, living situation, alcohol risk, physical activity, smoking, fall in last 12 months and multi-morbidity

*Abbreviations*: *CI* Confidence Interval, *Ref*. Reference category

### Interaction analyses

A significant interaction term was observed for physical HRQoL between fear of falling score and gender (*P* < 0.001) and fear of falling score and physical activity (*P* < 0.001). There were no other interaction effects for physical HRQoL (*P* < 0.05). For mental HRQoL, a significant interaction was observed between fear of falling score and country (*P* < 0.001). There were no other interaction effects for mental HRQoL (*P* < 0.05). (Suppl. Table 2).

Stratified analyses for gender indicated a beta for fear of falling score of β = -1.37 (95%CI -1.54 to -1.20) for males and β = -0.92 (95%CI -1.03 to -0.81) for females with regard to physical HRQoL (Suppl. Table 3). For physical activity and physical HRQoL, the beta for the stratum of physical activity once a week or less was β = -0.85 (95%CI -0.98 to -0.72), and for the stratum of physical activity more than once a week β = -1.31 (95%CI -1.44 to -1.17) (Suppl. Table 4).

With regard to mental HRQoL, stratified analyses per country indicated the following beta’s for fear of falling scores for participants from United Kingdom β = -0.41 (95%CI -0.60 to -0.22), Greece β = -0.57 (95%CI -0.84 to -0.31), Croatia β = -0.63 (95%CI -0.79 to -0.46), the Netherlands β = -0.52 (95%CI -0.88 to -0.15), and Spain β = -0.61 (95%CI -0.92 to -0.30) (Suppl. Table 5).

## Discussion

In this study, a negative association between fear of falling and physical and mental HRQoL, corrected for previous falls, was observed among community-dwelling older persons of 70 years and older in five European countries. The effect size between participants of the ‘low’ and ‘high’ fear of falling subgroups was large for both physical and mental HRQoL. Results concerning the prevalence of moderate and high fear of falling (29.6% and 20.3%, respectively) are comparable to prevalence numbers reported in other studies, although measurement tools to define prevalence numbers have differed [6, 7, 9–11]. Women in this study reported a higher prevalence of high fear of falling compared to men in our study, which is also in line with previous research [8, 32]. The negative association between fear of falling and physical and mental HRQoL observed in our study confirms previous studies' findings [8, 39]. Previous research showed that fear of falling often increases after experiencing a previous fall [15]. However, our findings indicate that the negative association with HRQoL holds even corrected for previous falls. (Data not shown.) While previous studies confirmed the negative impact of fear of falling mostly in specific subgroups, such as patients with chronic conditions [40, 41] or frail older persons [39], this study underlines the presence and need for support in a sample of community-dwelling older persons.

Cohen’s d effect size comparing participants of the ‘low’ and ‘high’ fear of falling subgroups for physical and mental HRQoL indicated a large effect for both with *d* ≥ 0.80 (Table 1). Moreover, participants who reported moderate fear of falling experienced significantly lower physical HRQoL compared to participants who reported low fear of falling. Physical HRQoL refers to an individual’s or a group’s perceived physical health over time [42]. Fear of falling may be associated with physical inactivity behavior among older persons [16]. This association is of concern as muscle strength and activity generally decline with older age [43]. Physical inactivity may add to this decline, increasing the risk of falling [44]. A Cochrane systematic review and meta-analysis has shown significant effects of physical activity promotion on reducing fear of falling [25]. We recommend preventive efforts to support older persons, especially those who experience fear of falling, to stay active to maintain and strengthen physical health [45]. Also, post-fall anxiety can further contribute to deconditioning, weakness and abnormal gait due to avoiding activity by being too careful [16]. Therefore, health professionals need to be aware of the associations between fear of falling, experiencing a fall, physical activity behaviors, physical health and physical HRQoL of older persons.

There was a significant interaction between fear of falling and gender in the association with physical HRQoL; in the male subgroup, the association between fear of falling and physical HRQoL was significant. In the literature, the crude prevalence of fear of falling was higher in females than in males [8, 38]. Our data support this difference; however, we did not test for this. These findings need to be confirmed by future studies, as also the sample size in the high fear of falling subgroups in men was low (*n* = 112). Another possible explanation for the interaction between gender and fear of falling, is that both the female gender and frailty have been reported to increase the risk of having fear of falling [46].

To our knowledge, this study is one of the first to report an association between fear of falling and lower mental HRQoL in community-dwelling older persons. Mental HRQoL reflects an individual’s or a group’s perceived mental health over time [42]. The negative association between fear of falling and mental health may be bidirectional. Fear of falling is associated with the emergence of depressive symptoms and impaired executive functioning; on the other hand, clinical depression may be associated with the emergence of redundant fear of falling [47, 48]. We recommend that future studies use longitudinal data to evaluate the direction of the association and the pathways between fear of falling and mental HRQoL. A randomized controlled trial performed among 415 community-dwelling older adults aged 60 years and over, both sexes, with a FES-I score of > 23, showed a significant reduction in fear of falling using cognitive behavioral therapy [49]. These type of interventions might potentially impact fear of falling and mental HRQoL.

The pathways between fear of falling and both mental and physical HRQoL might be influenced by different factors [50]. For example, activity avoidance might affect mental HRQoL because of less social interaction. While physical HRQoL is also affected by activity avoidance, but more on the pathway of loss of muscle strength. These factors can be important to tailor specific psychological or physical care for older persons who experience fear of falling to help them cope with challenges in their daily life [8, 51].

### Methodological considerations

A strength of this study is that we were able to analyze a large set of data from a general community-dwelling population across five European countries. The associations between fear of falling and physical and mental HRQoL were studied using validated measurement instruments (i.e. FES-I and SF-12) [19]. A diverse sample of older persons living independently in the community was studied, adding to the existing literature that mainly focused on subgroups such as frail older persons and persons with specific chronic conditions [40, 41]. The following methodological considerations should be taken into account. First, the study’s cross-sectional design does not allow for a causal interpretation of the relationship between fear of falling and HRQoL. Second, some selection bias might have occurred because of excluding persons who were not fluent in the local language. Third, all outcome measures were self-reported using validated questionnaires. Fourth, there is a big difference between the incidence of high fear of falling within countries; even while using validated questionnaires, such as the FES-I, differences in interpretability between countries cannot be ruled out fully. A possible explanation for this difference is that Croatia, within our study population, has the highest proportion of women, the highest proportion of people who exercise once or less a week, and the lowest physical HRQoL score. Fifth, only the main outcomes, physical and mental health-related quality of life, of the SF-12 were used as co-variates; not subdomains, like social functioning or physical role domains. We recommend future research to explore the associations between subdomains of health-related quality of life and falls. Finally, although correction for potential confounders was applied in the analyses, there may be unmeasured or residual confounding.

To conclude, this study showed an association between higher perceived levels of fear of falling and lower physical and mental HRQoL in a large European multi-country population of community-dwelling older persons. The findings emphasize the relevance for health professionals to assess and address fear of falling, recommend preventive programs to promote physical activity, reduce fear of falling, and maintain or increase physical strength and physical and mental HRQoL.

## Supplementary Information

**Additional file 1: Supplementary Figure 1.** Participants flow chart. **Supplementary Table 1.** Fear of Falling score and physical and mental Health-Related Quality of Life (*n*=2189). **Supplementary Table 2.** *P*-values and coefficients for interaction analyses for Fear of Falling score in the association with physical and mental HRQoL (n=2040). **Supplementary table 3.** The association between Fear of Falling score and physical HRQoL, stratified by gender (*n*=2040). **Supplementary table 4.** The association between Fear of Falling score and physical HRQoL, stratified by physical activity level (*n*=2040). **Supplementary table 5.** The association between Fear of Falling score and mental HRQoL, stratified by country (*n*=2040). **Supplementary Table 6.** Comparison of included and excluded participants (*n*=2325).

## Data Availability

The datasets generated and/or analyzed during the current study are not publicly available due to privacy concerns but are available from the corresponding author on reasonable request.

## Abbreviations

β: Beta
95% CI: 95% Confidence interval
ANOVA: One-way analysis of variance
AUDIT-C: Alcohol Use Disorder Identification Test-Consumption
HRQoL: Health-Related Quality of Life
ISCED: International Standard Classification of Education
MCS: Mental Component Summary of the SF-12
OR: Odds ratio
PCS: Physical Component Summary of the SF-12
SF-12: The 12-Item Short Form Health Survey
Short FES-I: Falls Efficacy Scale International Short
UHCE: Urban Health Centres Europe
