# Supplementary material for: The association of fear of falling and physical and mental Health-Related Quality of Life (HRQoL) among community-dwelling older persons; a cross-sectional study of Urban Health Centres Europe (UHCE)
Source: BMC Geriatr. 2023 May 13;23:291. doi: 10.1186/s12877-023-04004-y (PMC10182691; doi:10.1186/s12877-023-04004-y)
Supplement: Supplementary file 1 — Additional file 1: Supplementary Figure 1. Participants flow chart. Supplementary Table 1. Fear of Falling score and physical and mental Health-Related Quality of Life (n=2189). Supplementary Table 2. P-values and coefficients for interaction analyses for Fear of Falling score in the association with physical and mental HRQoL (n=2040). Supplementary table 3. The association between Fear of Falling score and physical HRQoL, stratified by gender (n=2040). Supplementary table 4. The association between Fear of Falling score and physical HRQoL, stratified by physical activity level (n=2040). Supplementary table 5. The association between Fear of Falling score and mental HRQoL, stratified by country (n=2040). Supplementary Table 6. Comparison of included and excluded participants (n=2325). [file 12877_2023_4004_MOESM1_ESM.docx]

# Supplementary Tables / Figures

Enrolled in UHCE at baseline (n=2325)

Available for analysis (n=2189)

Excluded from analysis (n=136)^a^
 no data available on:

- Age (n=136)
- Gender (n=136)
- HRQoL (SF-12, n=127)
- Fear of falling (Short FES-I, n=21)

^a^ Some participants met more than 1 criterion. Abbreviations: HRQoL = Health-related quality of life measured by the SF-12; SF-12 = Short Form health survey; Short FES-I = Falls Efficacy Scale International Short

## Supplementary Figure 1. Participants flow chart

## Supplementary Table 1. Fear of Falling score and physical and mental Health-Related Quality of Life (n=2189)

|  | | **Physical HRQoL**  **(adjusted model)** | | | **Mental HRQoL**  **(adjusted model)** | | | |
| --- | --- | --- | --- | --- | --- | --- | --- | --- |
|  | | Beta (95% CI) | *P*-value | Beta (95% CI) | *P*-value |  |  |  |
| Fear of falling score | | -0.422 (-1.130; -0.939) | < 0.001 | -0.339 (-0.839; -0.641) | < 0.001 | |  |  |
| *P*-values are based on multivariable linear regression models. Adjusted model: continuous fear of falling score, age, gender, country, level of education, living situation, alcohol risk, physical activity, smoking, fall in last 12 months and multi-morbidity. Abbreviations: HRQoL= Health-related quality of life measured by the SF-12; Short Form health survey; *P*-values are based on multivariable linear regression models. | | | | | | | | |

## Supplementary Table 2. *P*-values and coefficients for interaction analyses for Fear of Falling score in the association with physical and mental HRQoL (n=2040)

|  | **Physical HRQoL** | **Mental HRQoL** |
| --- | --- | --- |
| Items | *P*-value | *P*-value |
| Age * fear of falling score | 0.022 | 0.188 |
| Gender * fear of falling score | **<0.001** | 0.858 |
| Country * fear of falling score | 0.488 | **0.001** |
| Level of education * fear of falling score score | 0.388 | 0.552 |
| Living situation * fear of falling score | 0.225 | 0.601 |
| Alcohol risk * fear of falling score | 0.022 | 0.698 |
| Physical activity * fear of falling score | **<0.001** | 0.237 |
| Smoking * fear of falling score | 0.947 | 0.559 |
| Multi-morbidity * fear of falling score | 0.851 | 0.029 |
| Fall in last 12 months * fear of falling score | 0.017 | 0.039 |

Significant *P*-values in bold; Bonferroni correction for multiple testing was applied (P=0.05/18=0.0028). *P*-values were derived by separately adding the interaction terms to the multivariable linear regression models adjusted for socio-demographic characteristics. Abbreviations: HRQoL = Health related quality of life;

## Supplementary table 3. The association between Fear of Falling score and physical HRQoL, stratified by gender (n=2040)

|  | Male  (n=793) |  | Female  (n=1247) |  |
| --- | --- | --- | --- | --- |
|  | Beta (95%CI) | p-value | Beta (95%CI) | p-value |
| FoF | -1.33 (-1.51; -1.16) | < 0.001 | -0.92 (-1.04; - 0.81) | < 0.001 |

Abbreviations: 95%CI=95% Confidence Interval; FoF, Fear of falling measure, Short FES-I continuous. Beta regression coefficients based on multivariable linear regression models with continuous fear of falling, age, country, level of education, living situation, alcohol risk, physical activity, smoking, fall in last 12 months and multi-morbidity.

## Supplementary table 4. The association between Fear of Falling score and physical HRQoL, stratified by physical activity level (n=2040)

|  | *=< once a week*  *(n=586)* | | *> once a week*  *(n=1454)* | |
| --- | --- | --- | --- | --- |
|  | Beta (95%CI) | p-value | Beta (95%CI) | p-value |
| FoF | -0.85 (-0.99; - 0.72) | < 0.001 | -1.30 (-1.44; -1.16) | < 0.001 |

Abbreviations: 95%CI=95% Confidence Interval; FoF, Fear of falling measure, Short FES-I continuous. Beta regression coefficients based on multivariable linear regression models with continuous fear of falling, age, gender, country, level of education, living situation, alcohol risk, smoking, fall in last 12 months and multi-morbidity.

## Supplementary table 5. The association between Fear of Falling score and mental HRQoL, stratified by country (n=2040)

|  | *United Kingdom*  *(n=530)* | | *Greece*  *(n=269)* | | *Croatia*  *(n=440)* | | *The Netherlands*  *(n=318)* | | *Spain*  *(n=483)* | |
| --- | --- | --- | --- | --- | --- | --- | --- | --- | --- | --- |
|  | Beta (95%CI) | p-value | Beta (95%CI) | p-value | Beta (95%CI) | p-value | Beta (95%CI) | p-value | Beta (95%CI) | p-value |
| FoF | -0.41  (-0.61; -0.22) | < 0.001 | -0.53  (-0.80; -0.25) | < 0.001 | -0.64  (-0.81; -0.47) | < 0.001 | -0.48  (-0.85; -0.11) | 0.014 | -0.51  (-0.83; -0.20) | 0.001 |
| Abbreviations: 95%CI=95% Confidence Interval; FoF, Fear of falling measure, Short FES-I continuous. Beta regression coefficients based on multivariable linear regression models with continuous fear of falling, age, country, level of education, living situation, alcohol risk, physical activity, smoking, fall in last 12 months and multi-morbidity. | | | | | | | | | | |

## Supplementary Table 6. Comparison of included and excluded participants (n=2325)

|  | Included  (N=2189) | Excluded  (N=136) | *P*-value |
| --- | --- | --- | --- |
|  |  |  |  |
| **Age (SD)** | 79.7 (5.6) | 80.5 (6.5) | **0.017** |
| **Gender** |  |  | 0.270 |
| Female (%) | 1326 (60.6) | 73 (55.7) |  |
| Male (%) | 863 (39.4) | 58 (44.3) |  |
| **Country** |  |  | **<0.001** |
| United Kingdom (%) | 536 (24.5) | 29 (21.3) |  |
| Greece (%) | 334 (15.3) | 42 (30.9) |  |
| Croatia (%) | 485 (22.2) | 14 (10.3) |  |
| The Netherlands (%) | 339 (15.5) | 46 (33.8) |  |
| Spain (%) | 495 (22.6) | 5 (3.7) |  |
| **Level of education** |  |  | 0.103 |
| Primary or less (%) | 593 (27.4) | 40 (30.8) |  |
| Secondary or equivalent (%) | 1357 (62.7) | 71 (54.6) |  |
| Tertiary or higher (%) | 213 (9.8) | 19 (14.6) |  |
| **Living situation** |  |  | 0.103 |
| Living with others (%) | 1358 (62.2) | 71 (55.0) |  |
| Living alone (%) | 825 (37.8) | 58 (45.0) |  |
| **Alcohol risk** |  |  | 0.085 |
| No (%) | 1538 (73.8) | 80 (67.7) |  |
| Yes (%) | 546 (26.2) | 40 (33.3) |  |
| **Physical activity** |  |  | 0.852 |
| < once a week (%) | 617 (28.4) | 37 (29.1) |  |
| >= once a week (%) | 1558 (71.6) | 90 (70.9) |  |
| **Smoking** |  |  | **0.003** |
| Yes (%) | 158 (7.2) | 19 (14.2) |  |
| No (%) | 2026 (92.8) | 115 (85.8) |  |
| **Multi-morbidity** |  |  | 0.754 |
| Yes (%) | 1988 (90.9) | 121 (91.7) |  |
| No (%) | 200 (9.1) | 11 (8.3) |  |
| **Fall in last 12 months** |  |  | 0.153 |
| Yes (%) | 1506 (69.3) | 83 (63.4) |  |
| No (%) | 667 (30.7) | 48 (36.6) |  |
| **HRQoL** |  |  |  |
| PCS-12 (SD) | 41.8 (12.1) | 43.0 (14.9) | 0.342 |
| MCS-12 (SD) | 50.2 (10.7) | 46.3 (8.0) | 0.100 |
| **Fear of falling** |  |  |  |
| Short FES-I score (SD) | 10.6 (4.9) | 10.9 (4.8) | 0.875 |

Notes: SD = standard deviation, PCS-12 = PCS-12 = 12-item Physical Component Summary, with higher scores indicating higher levels of health; MCS-12 = 12-item Mental Component Summary, with higher scores indicating higher levels of health; *P*-values are based on *t*-tests for continuous variables (age, PCS-12, MCS-12 and Short FES-I) and Pearson’s chi-squared tests for categorical variables (all other variables).
